# Syncope, Fear of Falling and Quality of Life Among Older Adults: Findings From the Irish Longitudinal Study on Aging (TILDA)

**DOI:** 10.3389/fcvm.2020.00007

**Published:** 2020-02-07

**Authors:** Kevin McCarthy, Mark Ward, Román Romero Ortuño, Rose Anne Kenny

**Affiliations:** ^1^The Irish Longitudinal Study on Ageing (TILDA), Department of Medical Gerontology, Trinity College Dublin, Dublin, Ireland; ^2^Mercer's Institute for Successful Ageing, St. James's Hospital, Dublin, Ireland

**Keywords:** syncope, quality of life, TILDA, CASP-12, fear of falling (FOF)

## Abstract

**Objective:** Syncope is a prevalent condition that has a marked impact on quality of life. We examined the association between syncope and quality of life (QoL) and whether this association was explained by fear of falling (FoF).

**Methods:** We examined data from Wave 3 of The Irish Longitudinal Study on Aging (TILDA), of adults aged ≥50 years (*n* = 4,946) who were asked to report syncope and who completed the CASP-12 QoL instrument. Analyses were stratified by age and gender.

**Results:** Over 20% of participants reported having a previous syncopal episode, while 8% reported a faint, blackout or unexplained fall in the last year. QoL scores decreased as the burden of syncope increased: linear regression models adjusted for covariates showed that those having had two or more syncopal episodes in the last year reported a significantly lower CASP-12 score compared to those with none (*p* = 0.011). FoF partially mediated the association between syncope and QoL, particularly among younger participants.

**Conclusions:** Syncope is a common condition among older adults that has a deleterious effect on QoL, with ≥2 recent syncopal episodes having a particularly adverse impact on QoL. FoF is a potential pathway which may both explain this association and allow therapeutic interventions by health practitioners.

## Introduction

Syncope has been defined by the European Society of Cardiology as a transient loss of consciousness due to transient global cerebral hypoperfusion, characterized by rapid onset, short duration and followed by a spontaneous and complete recovery (1). All forms of syncope are thought to occur due to a sudden decrease of cerebral blood flow but there are numerous mechanisms for this, ranging from the benign, such as vasovagal syncope (VVS), whereby cerebral hypoperfusion is induced by reflex bradycardia and/or peripheral vasodilatation, to potentially life-threatening causes such as those involving an underlying cardiac dysrhythmia (2). The causes range in prevalence from 60% reflex to 15% cardiac with treatments varying depending on the underlying cause (3). Other causes of syncope include, but are not limited to, orthostatic hypotension and structural heart disease. The cause of syncope may be multifactorial or indeed may not be identified at all, however, more recently, with the advent of newer technologies to aid investigations, rates of unexplained syncope have reduced to between 14 and 17.5% (4, 5).

Increasing incidence of syncope with advancing age can be accounted for by gradual age-related physiological impairments, namely “neuro-cardiovascular instability”—the regulation of heart rate, blood pressure, and cerebral blood flow (6), particularly with postural changes, in combination with increasing incidence of co-morbidities and polypharmacy that impair cardiovascular compensation (7).

Public policy is increasingly focused on the issue of successful aging in the hope of enabling individuals to lead enjoyable lives into old age. Quality of life (QoL) is an important feature of successful aging and it is vital that we better understand the health and related factors that affect QoL. Chronic disease and co-morbidity are features of aging (8) and their prevalence has increased along with life expectancy. This has been shown to be deleterious to QoL as people age (9). Less is known about the associations between individual conditions and QoL and how QoL is affected; for instance, through physical symptoms, functional limitations, or psychological effects, any of which could negatively impact upon physical and mental abilities, mood and social participation. It has been shown that a variety of factors including demographics, health, and social characteristics are associated with changes in QoL over time and that QoL is not just a function of aging or declining health but influenced by factors such as loneliness and social participation (10) which themselves may or may not be impacted by ill health. Improving our understanding of the different factors that influence QoL in the context of co-morbidity is vital in optimizing successful aging.

While maximizing QoL is an accepted aim for individuals and on a population level, a consensus is lacking on how best to define or measure QoL. Many measures of QoL include indicators of physical health and function which can make it more difficult to disentangle the associations between chronic disease and QoL, especially in older populations where chronic disease and co-morbidity are more prevalent and where health has often been used as a proxy for QoL rather than being simply a factor that influences it. The CASP-19 measure uses a broader definition of QoL, specifically developed for use with older people, consisting of 19 Likert-scaled items, encompassing four domains; control, autonomy, self-realization, and pleasure, the initials of which make up its acronym. It aims to assess the degree to which human needs are satisfied and can be affected by multiple factors including, but not limited to, health as measure of QoL (11) and has been shown to be a useful scale for measuring QoL in older people (12).

The QoL in syncope patients has largely been studied only in small groups, mainly in referral centers. Syncope has been shown to have moderate to severe effects on QoL from both a health and functional point of view, especially in patients with a recent onset of symptoms, with 33% having reported functional impairments with daily activities, which was clinically as well as statistically significantly worse than the reference population (13). QoL associated with recurrent syncope has been reported as equivalent to chronic conditions such as severe rheumatoid arthritis and chronic lower back pain (14) with 76% reporting that syncope interfered with their life or activities of daily living, with driving and employment also frequently affected. Syncope, even benign VVS, can result in not only loss of consciousness but also postural tone, which depending on one's occupation may be very hazardous—for instance when driving a truck/bus, working at heights or in proximity to flames or hot/dangerous materials/machinery. Indeed, even pre-syncope may result in falls or occupational accidents. Complexities around identifying causes and potential diagnoses may result in undue caution and unjustified delays in returning to work or driving (15). Syncope, regardless of one's occupation, can result in physical injuries and can be fatal, with those suffering from syncope experiencing increased healthcare utilization, unwanted change in employment status, driving restrictions, decreased social participation, and subjective decrease in self-worth, all of which impair QoL.

While one study found that syncope leads to a greater psychosocial than physical impairment (14) another found a greater impact of physical impairment than psychosocial (16). Whereas, both groups recruited participants from a syncope referral center, Linzer's group was smaller, older, more predominantly female and had a higher frequency of syncopal episodes compared to Rose's (62 participants vs. 136, mean age 49 ± 19 vs. 40 ± 17, 29% male vs. 42%, median 10 syncopal episodes in median 11 months vs. median 7 in 60). Different QoL measurement tools were used also, the strengths and weaknesses of which were discussed in detail in Rose's paper. Is it possible that age and/or sex may influence the associations between syncope and QoL?

Fear of falling (FoF) has been found to be associated with recent experience of falls and decreased QoL (17), lower activity levels, fewer social contacts (18), and reduced functional capacity (19). Those with severe FoF were also less likely to include leisure activities into their daily lives (20). FoF is associated with poor health and institutionalization (21). When 12 studies were summarized syncope was found to account for <1% of all causes of falls, however dizziness and vertigo, some of which may be attributable to pre-syncope, account for a mean of 13% (22). Even though syncope is a known cause of falls and impaired QoL, and that FoF is also known to have a detrimental effect on functional status, social participation and QoL there is very little known about the relationship between syncope and FoF and how it relates to QoL. Is FoF a mechanism through which syncope affects QoL or is it independent of it? Any intervention to reduce FoF in the context of syncope could potentially improve aspects of social participation, functional limitations, and may even improve rates of institutionalization.

This study sought to investigate the relationship between syncope and QoL in an older community-dwelling population, compared to findings previously observed in small samples of participants recruited from specialist referral centers. In addition, we aimed to further investigate this relationship by taking into account features of the syncopal history such as timing (recent vs. distant) and frequency (number of syncopal events). We also aimed to investigate possible age and/or sex differences and explored the possible role of FoF in mediating the relationship between syncope and QoL.

## Methods

### Sample

This observational study is based on data from the third wave of The Irish Longitudinal Study of Aging (TILDA), a prospective study of the health, social and economic circumstances and representative of community-dwelling adults aged ≥50 in Ireland. The third wave of data was collected between 2014 and 2015 with the data collection process being described in detail elsewhere (23, 24). As part of the study participants completed a Computer Assisted Personal Interview (CAPI), conducted in participants' homes by trained interviewers and a paper-based Self-Completion Questionnaire (SCQ) completed privately by participants at each wave. Those under the age of 50 were excluded.

### Outcome Variable—QoL

QoL was measured as part of the SCQ where participants were asked to complete the CASP-12 instrument, a revised version of the CASP-19 QoL scale which has been shown to be a useful scale for measuring QoL in older people (12), has been recommended for use ahead of the CASP-19 as it has been shown to have stronger measurement properties than the original CASP-19 (25) and it has been shown to be psychometrically valid in TILDA (26). Participants rated how often they feel a particular way about their life, on 12 Likert-scaled items, ranging from often to never, with regards to the four domains of QoL measured by CASP; control, autonomy, self-realization, and pleasure, with possible scores ranging from 0 to 36 with a higher score indicating a better QoL (Table 1). CASP-12 was the outcome variable in this study.

**Table 1:** Measures of control, autonomy, self-realization and pleasure in the CASP-12.

**Scale**	**Description**	**Item**	**Score range**
Control	Ability to actively participate in one's environment	My age prevents me from doing the things I would like to I feel that what happens to me is out of my control I feel free to plan for the future I feel left out of things	0–12
Autonomy	Right of the individual to be free from the unwanted interference of others	I feel that I can please myself in what I can do My health stops me from doing things I want to do Shortage of money stops me doing things I want to do	0–9
Self-realization	Fulfillment of one's potential	I feel satisfied with the way my life has turned out I feel life is full of opportunities	0–6
Pleasure	Sense of happiness or enjoyment derived from engaging with life	I look forward to each day I feel that my life has meaning I enjoy being in the company of others	0–9

### Main Independent Variables—Syncope and FoF

A section of the CAPI asked participants “Have you ever had a blackout or fainted?” or “Since your last interview, have you had a blackout or fainted?” Those who had reported a positive response to the equivalent question in the CAPI from wave 2 were fed forward and given a positive response. Those who had not answered the question in wave 2 or who had not previously answered positively were given the options yes, no, don't know or refuse to answer. Those who answered yes were added to those fed forward from wave 2 and these participants were classed as having had “previous syncope.” Previous syncope is the first predictor variable and includes previous blackouts and faints.

Another question asked, “Have you fallen in the last year/since your last interview?” with options being yes, no, don't know or refuse to answer. Those who answered yes were instructed to continue to “How many times have you fallen in the last year” and then “Was this fall/were any of these falls non-accidental, i.e., with no apparent or obvious reason?” with the same set of options available for responses as before. Those who answered yes were classified as having had an “unexplained fall in the last year.”

Participants who had a positive response to “Since your last interview, have you had a blackout or fainted?” were asked “Approximately how many times have you had a blackout or fainted in the last year?” while those participants who were fed forward with a positive response to the equivalent question from wave 2 were asked the same question. Those participants who answered anything ≥1 were classified as having had a “blackout or faint in the last year/since last interview”. Those participants were added to any with an “unexplained fall in the last year” and combined were classified as having had a syncopal episode in the last year or “recent syncope.” Recent syncope is the second predictor variable and includes faints, blackouts, and unexplained falls.

Participants who were asked “Approximately how many times have you had a blackout or fainted in the last year?” and answered ≥1 were classified as having had a “blackout or faint in the last year” with participants categorized as having none, one, or two or more recent blackouts or faints or “recurrent syncope in the last year.” Recurrent syncope in the last year is the third predictor variable and includes blackouts and faints but not unexplained falls as this could not be quantified separately to any type of fall. Those participants who reported any unexplained fall in the last year but not a faint or blackout were excluded from the “none” category for recent blackout or faint.

In the CAPI, participants, regardless of responses to previous questions about falls, were asked “Are you afraid of falling?” Those who responded positively were defined as having “FoF.” FoF is the fourth predictor variable.

### Covariates

Socioeconomic characteristics including age, sex, education and marital status were recorded.

History of chronic disease was obtained through a self-reported doctor-diagnosed history of diabetes and cardiovascular disease (heart attack, heart failure, stroke, Transient Ischemic Attack, atrial fibrillation, hypertension, and/or angina). The level of non-cardiovascular physical co-morbidity was categorized as 0, 1, or ≥2 self-reported doctor-diagnosed chronic conditions including cataracts, glaucoma, age-related macular degeneration, chronic lung disease, asthma, arthritis, osteoporosis, cancer, Parkinson's disease, peptic ulcer disease, venous ulcers, liver disease, thyroid disease, kidney disease, anemia, or any type of incontinence. Chronic disease has been shown to affect QoL, directly and indirectly, through increased deficits in physical function and activity (11) while self-reported doctor diagnoses have been shown to be a reliable measure of disease burden compared to medical records (27).

The total number of regular prescribed medications was recorded and coded using the World Health Organization Anatomical Therapeutic Chemical index with those participants taking 5 or more regular medications excluding supplements being defined as being subject to “polypharmacy.” Smoking status was also recorded.

In terms of non-physical issues that may affect QoL, a self-reported history of having suffered either childhood physical or sexual abuse was obtained, as was loneliness and depression, all of which have been shown to have a negative effect on QoL (28). Apart from the impact it may have on later life QoL childhood trauma has been shown to have an association with increased cardiovascular risk in mid-life (29) and has also been linked with a lifelong tendency for syncope (30). The degree of loneliness or social isolation participants perceived was recorded using the University of California Los Angeles (UCLA) loneliness scale, on 5 Likert-scaled items ranging from hardly ever or never to often with scores summed to create a range from 0 to 10 with higher scores indicating greater levels of loneliness (31). A current or recent depressive disorder was measured using the Composite International Diagnostic Interview-Short Form (CIDI-SF) whereby participants dichotomously did or did not fulfill criteria for a major depressive episode in previous 12 months. CIDI-SF has been shown to correctly classify CIDI cases of major depressive episodes with an accuracy of 93% and generalized anxiety disorder with an accuracy of >99% (32).

### Statistical Analysis

Stata/MP 14.1 software was used for all statistical analysis (33).

Ordinary Least Squares equal-interval scale linear regression models were used to examine the associations between syncope (previous, recent, and recurrent), QoL and FoF.

Cross-sectional weights were applied to account for attrition between waves and to adjust the sample making it more representative of the population according to age, sex, education, and location compared to known census data compiled by the Central Statistics Office.

Separate models were run with groups stratified by sex and age (<75 and ≥75) in a step-wise manner, initially adjusted for the covariates age, age squared (to account for the non-linear relationship observed between age and CASP score) (28), sex, marital status, and education (Model 1), before being further adjusted for smoking status, cardiovascular disease, diabetes, chronic disease, polypharmacy, history of childhood physical or sexual abuse, loneliness, and depression (Model 2). Finally, the models were adjusted for FoF (Model 3) to examine its effect and relative importance.

## Results

There were 6,454 participants ≥50 years of age who completed the CAPI. Of these participants, 76.6% (*n* = 4,946) had complete CASP-12 data, of which 2.1% (*n* = 103) were missing data for any of the variables of interest, with those participants excluded, resulting in a final sample of 4,933 participants for model 1 and 4,843 for models 2 and 3. These 4,843 participants were characterized overall before being stratified according to sex and age.

Over one in five (21.7%, SE: 0.767, 95% CI: 20.27–23.29) reported a previous syncopal episode while nearly one in twelve (8.07%, SE: 0.471, 95% CI: 7.19–9.04) reported a recent syncopal episode. Both previous and recent syncope were more commonly observed among women with the incidence of recent syncope being higher among those 75 or older for both sexes (Table 2).

**Table 2:** Distribution of key characteristics of the 4,843 participants included in Model 2 and 3 with standard errors and confidence intervals.

			**Std. Err**.	**95% CI**	***N***
Gender	Male	45.87%	0.623	44.65–47.10	4,843
	Female	54.13%	0.623	52.90–55.35	
Mean age (years)	Overall	64.83	0.196	64.44–65.21	4,843
	Male	65.20	0.218	64.77–65.63	2,172
	Female	64.51	0.246	64.03–65.00	2,671
	Male≥75	79.88	0.221	79.44–80.31	430
	Female≥75	80.26	0.210	79.85–80.68	473
	Male <75	62.18	0.161	61.87–62.50	1,742
	Female <75	60.73	0.175	60.38–61.07	2,198
Highest level of education obtained	Primary or below	28.38%	0.913	26.62–30.20	4,843
	Secondary	46.75%	0.855	45.08–48.43	
	Tertiary or above	24.87%	0.801	23.33–26.48	
Marital status	Married	72.33%	0.812	70.71–73.90	4,843
	Never married	7.77%	0.467	6.90–8.74	
	Separated/divorced	6.97%	0.422	6.18–7.84	
	Widowed	12.93%	0.554	11.88–14.06	
Level of co-morbidity	0 chronic conditions	39.62%	0.843	37.98–41.29	4,843
	1 chronic condition	31.11%	0.749	29.66–32.59	
	≥2 chronic conditions	29.27%	0.793	27.74–30.85	
Proportion who had a recent syncopal event	Overall	8.07%	0.471	7.19–9.04	4,843
	Male	6.13%	0.557	5.13–7.32	2,172
	Female	9.70%	0.676	8.45–11.11	2,671
	Male≥75	11.35%	1.684	8.43–15.11	430
	Female≥75	16.57%	1.872	12.20–20.58	473
	Male <75	5.06%	0.575	4.04–6.32	1,742
	Female <75	8.05%	0.685	6.81–9.50	2,198
Proportion who had previous syncopal event	Overall	21.74%	0.767	20.27–23.29	4,843
	Male	19.22%	0.965	17.39–21.19	2,172
	Female	23.88%	1.041	21.90–25.98	2,671
	Male≥75	16.11%	1.970	12.59–20.37	430
	Female≥75	25.75%	2.274	21.53–30.47	473
	Male <75	19.86%	1.069	17.84–22.04	1,742
	Female <75	23.43%	1.125	21.29–25.71	2,198
Mean CASP-12 score	Overall	26.73	0.095	26.55–26.92	4,843
	Male	26.76	0.132	26.50–27.02	2,172
	Female	26.71	0.127	26.46–26.96	2,671
	Male≥75	26.55	0.255	26.05–27.06	430
	Female≥75	25.82	0.257	25.31–26.32	473
	Male <75	26.80	0.152	26.51–27.10	1,742
	Female <75	26.93	0.145	26.64–27.21	2,198

There was no significant difference on CASP-12 scores between the “overall” group (Mean CASP-12 score 26.73, SE: 0.095, 95% CI: 26.55–26.92) and either sex when grouped as total number for each sex regardless of age. When stratified by age <75 or ≥75, younger females (26.93, SE: 0.145, 95% CI: 26.64–27.21) had better QoL than older (25.82, SE: 0.257, 95% CI: 25.31–26.32). There was no difference for men regardless of age group.

Of the 6,454 participants ≥50 years of age included in the study who completed the CAPI 4,072 had complete CASP-12 data, relevant covariates, as well as information relating to the number of recent blackouts or faints or “recurrent syncope” (once those who reported any unexplained fall but not a faint or blackout were excluded). Table 3 shows characteristics of the different categories for recurrent syncope in the last year.

**Table 3:** Distribution of key characteristics of the 4,072 participants included in Model 2R and 3R with standard errors and confidence intervals.

	**Number of faints or blackouts in past year**		**Std. Err**.	**95% CI**	***N***
Proportion of total males (%)	0	96.94%	4.427	95.94–97.70	1,872
	1	1.95%	3.343	1.39–2.72	
	≥2	1.11%	2.631	0.70–1.77	
Proportion of total females (%)	0	95.92%	4.966	94.82–96.79	2,200
	1	2.91%	4.135	2.20–3.85	
	≥2	1.17%	2.967	0.71–1.92	
Mean age (male) (years)	0	64.95	0.235	64.48–65.41	1,812
	1	67.62	1.747	64.08–71.17	39
	≥2	66.85	1.687	63.30–70.39	21
Mean age (female) (years)	0	63.92	0.262	63.41–64.44	2,117
	1	67.47	1.799	63.87–71.08	60
	≥2	68.24	2.953	62.04–74.44	23
Mean CASP12 Score (male)	0	27.00	0.144	26.71–27.28	1,812
	1	25.55	1.225	23.06–28.03	39
	≥2	22.53	1.452	19.48–25.58	21
Mean CASP12 score (female)	0	26.88	0.144	26.60–27.17	2,117
	1	25.51	1.050	23.41–27.62	60
	≥2	23.76	1.078	21.50–26.03	23

There was no significant association between previous syncope and QoL, overall or in any of the sex and age stratified groups.

Results for the weighted linear regression models showing associations of QoL with recent syncope are summarized in Tables 4–6 showing Models 1, 2, and 3, respectively.

**Table 4:** Model 1—weighted linear regression model showing associations of QoL with recent syncope adjusted for age, age-squared, sex, marital status, and education.

**Category (*n*)**	**Coefficient (95% CI)**	**Std. Err**.	***P*-value**	***R*^**2**^**
Overall (4,933)	−2.31 (−3.01, −1.61)	0.355	<0.001	0.051
Male (2,213)	−2.90 (−4.00, −1.79)	0.561	<0.001	0.059
Female (2,720)	−1.97 (−2.84, −1.09)	0.446	<0.001	0.047
Male ≥ 75 (448)	−3.23 (−4.76, −1.69)	0.779	<0.001	0.076
Female ≥ 75 (484)	−1.50 (−2.81, −0.19)	0.668	0.025	0.035
Male <75 (1,765)	−2.68 (−4.11, −1.24)	0.730	<0.001	0.059
Female <75 (2,236)	−2.18 (−3.29, −1.07)	0.564	<0.001	0.048

**Table 5:** Model 2—weighted linear regression model showing associations of QoL with recent syncope adjusted for Model 1 covariates plus cardiovascular disease, diabetes, chronic health conditions, smoking, polypharmacy, history of childhood physical abuse, history of childhood sexual abuse, depression, and loneliness.

**Category (*n*)**	**Coefficient (95% CI)**	**Std. Err**.	***P*-value**	***R*^**2**^**
Overall (4,843)	−0.95 (−1.50, −0.41)	0.277	0.001	0.381
Male (2,172)	−1.72 (−2.57, −0.87)	0.433	<0.001	0.378
Female (2,671)	−0.45 (−1.13, 0.23)	0.346	0.190	0.392
Male ≥ 75 (430)	−1.81 (−2.98, −0.64)	0.593	0.002	0.342
Female ≥ 75 (473)	−1.15 (−2.12, −0.17)	0.495	0.021	0.360
Male <75 (1,742)	−1.78 (−2.88, −0.69)	0.557	0.001	0.391
Female <75 (2,198)	−0.18 (−1.11, 0.74)	0.469	0.694	0.405

**Table 6:** Model 3—weighted linear regression model showing associations of QoL with recent syncope adjusted for Model 2 covariates plus FoF.

**Category (*n*)**	**Coefficient (95% CI)**	**Std. Err**.	***P*-value**	***R*^**2**^**
Overall (4,843)	−0.81 (−1.36, −0.27)	0.277	0.004	0.386
Male (2,172)	−1.63 (−2.47, −0.79)	0.428	<0.001	0.380
Female (2,671)	−0.27 (−0.95, 0.40)	0.345	0.426	0.400
Male ≥ 75 (430)	−1.77 (−2.93, −0.62)	0.589	0.003	0.344
Female ≥ 75 (473)	−0.96 (−1.97, 0.04)	0.510	0.060	0.371
Male < 75 (1,742)	−1.66 (−2.75, −0.58)	0.552	0.003	0.393
Female < 75 (2,198)	−0.01 (−0.92, 0.91)	0.464	0.990	0.412

Fully adjusted weighted linear regression including the participants for whom all data was complete (*n* = 4,843) showed that recent syncope had a significant association with QoL (β = −0.95, SE: 0.277, 95% CI: −1.50 to −0.41, *p* = 0.001). This significance was maintained when adjusted for FoF (β = −0.81, SE: 0.277, 95% CI: −1.36 to −0.27, *p* = 0.004) and while the negative effect of syncope on QoL was reduced by FoF, it remained negative with the coefficient reducing from −0.95 to −0.81.

When stratified by sex, the association between recent syncope remained significant for males (β = −1.63, 95% CI: −2.47 to −0.79, *p* < 0.001) but not females (β = −0.27, 95% CI: −0.95 to 0.40, *p* = 0.426).

When stratified by age (but not sex), there was a significant association between recent syncope and QoL for both groups, with FoF having a greater impact on the reduction in the coefficient for those <75 (β = −0.81, 95% CI: −1.52 to −0.09, *p* = 0.027 to β = −0.65, 95% CI: −1.37 to 0.06, *p* = 0.073) than the older group (β = −1.33 95% CI: −2.12 to −0.53, *p* = 0.001 to β = −1.21, 95% CI: −2.02 to −0.40, *p* = 0.003) with a reduction in coefficient of 19.8 vs. 8.7%. While significance was maintained for the ≥75 group when adjusted for FoF this significance was lost when adjusted for FoF for those <75.

A significant association between recent syncope and QoL was observed for males both <75 and ≥75. Males <75 had a lesser negative coefficient (β = −1.66, 95% CI: −2.75 to −0.58, *p* = 0.003) compared with the older male group (β = −1.77, 95% CI: −2.93 to −0.62, *p* = 0.003) with FoF again accounting for a larger reduction as a proportion in the size of the coefficient for the younger group than the older (reduction in coefficient of 6.7 vs. 2.2%).

There was no significant association between recent syncope and QoL observed for women <75, whereas there was for women ≥75 (β = −1.15, 95% CI: −2.12 to −0.17, *p* = 0.021), however its statistical significance was lost once adjusted for FoF (β = −0.96, 95% CI: −1.97 to 0.04, *p* = 0.06).

**Figure 1:**
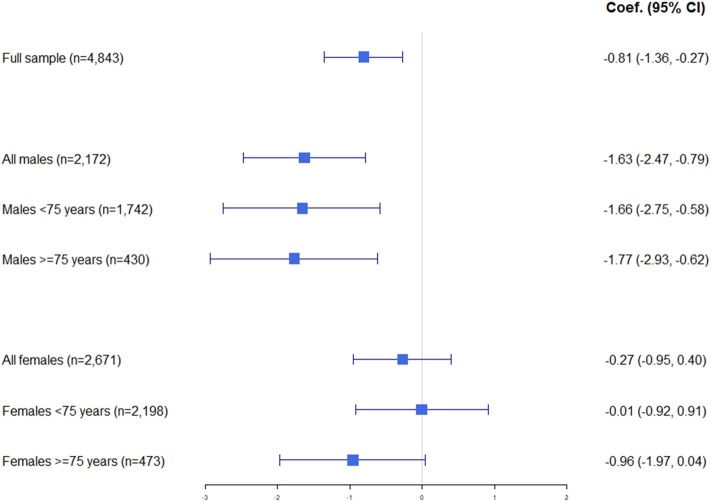
Graphical summary of results of weighted linear regression Model 3 with coefficient and 95% confidence intervals.

The results for Model 3 are summarized graphically in Figure 1 while results for the weighted linear regression models showing associations of QoL with recurrent syncope are summarized in Tables 7–9 showing Models 1R, 2R, and 3R, respectively.

**Table 7:** Model 1R—Weighted linear regression model showing associations of QoL with recurrent syncope adjusted for age, age-squared, sex, marital status, and education.

**Category (*n*)**	**Number of episodes**	**Coefficient (95% CI)**	**Std. Err**.	***P*-value**	***R*^**2**^**
Overall (4,146)	1	−1.09 (−2.60, 0.42)	0.768	0.156	0.047
	≥2	−3.05 (−4.65, −1.46)	0.812	<0.001	
Male (1,903)	1	−1.45 (−3.89, 1.00)	1.243	0.245	0.052
	≥2	−3.59 (−6.37, −0.81)	1.416	0.011	
Female (2,243)	1	−0.86 (−2.76, 1.03)	0.965	0.371	0.043
	≥2	−2.62 (−4.50, −0.75)	0.955	0.006	
Male ≥ 75 (377)	1	−3.33 (−6.95, 0.30)	1.840	0.072	0.053
	≥2	−2.71 (−5.91, 0.50)	1.627	0.097	
Female ≥ 75 (371)	1	1.62 (−0.01, 3.24)	0.824	0.050	0.029
	≥2	−1.80 (−6.26, 2.65)	2.263	0.426	
Male <75 (1,526)	1	−0.40 (−3.54, 2.75)	1.600	0.804	0.056
	≥2	−3.69 (−6.93, −0.46)	1.647	0.025	
Female <75 (1,872)	1	−1.83 (−4.38, 0.71)	1.297	0.158	0.049
	≥2	−2.89 (−4.52, −1.26)	0.831	0.001	

**Table 8:** Model 2R—weighted linear regression model showing associations of QoL with recurrent syncope adjusted for Model 1R covariates plus cardiovascular disease, diabetes, chronic health conditions, smoking, polypharmacy, history of childhood physical abuse, history of childhood sexual abuse, depression, and loneliness.

**Category (*n*)**	**Number of episodes**	**Coefficient (95% CI)**	**Std. Err**.	***P*-value**	***R*^**2**^**
Overall (4,072)	1	0.17 (−0.87, 1.21)	0.530	0.744	0.384
	≥2	−1.95 (−3.31, −0.58)	0.695	0.005	
Male (1,872)	1	0.28 (−1.21, 1.76)	0.756	0.714	0.376
	≥2	−3.82 (−6.13, −1.51)	1.177	0.001	
Female (2,200)	1	0.11 (−1.29, 1.51)	0.713	0.875	0.401
	≥2	−0.40 (−1.72, 0.93)	0.675	0.558	
Male ≥ 75 (362)	1	−1.67 (−3.37, 0.03)	0.865	0.055	0.342
	≥2	−2.12 (−4.81, 0.58)	1.369	0.123	
Female ≥ 75 (364)	1	1.08 (−0.80, 2.97)	0.957	0.260	0.378
	≥2	−0.70 (−4.07, 2.67)	1.710	0.683	
Male <75 (1,510)	1	0.94 (−1.03, 2.90)	0.999	0.350	0.391
	≥2	−4.35 (−6.94, −1.77)	1.316	0.001	
Female <75 (1,836)	1	−0.23 (−2.10, 1.64)	0.954	0.810	0.412
	≥2	−0.03 (−1.71, 1.66)	0.857	0.975	

**Table 9:** Model 3R—Weighted linear regression model showing associations of QoL with recurrent syncope adjusted for Model 2R covariates plus FoF.

**Category (*n*)**	**Number of episodes**	**Coefficient (95% CI)**	**Std. Err**.	***P*-value**	***R*^**2**^**
Overall (4,072)	1	0.24 (−0.82, 1.30)	0.540	0.659	0.390
	≥2	−1.82 (−3.22, −0.42)	0.712	0.011	
Male (1,872)	1	0.36 (−1.15, 1.87)	0.768	0.638	0.379
	≥2	−3.71 (−6.05, −1.37)	1.191	0.002	
Female (2,200)	1	0.15 (−1.27, 1.56)	0.722	0.840	0.410
	≥2	−0.26 (−1.59, 1.07)	0.678	0.704	
Male ≥ 75 (362)	1	−1.58 (−3.30, 0.13)	0.871	0.071	0.345
	≥2	−1.97 (−4.84, 0.90)	1.457	0.177	
Female ≥ 75 (364)	1	1.17 (−0.77, 3.11)	0.987	0.237	0.391
	≥2	−0.63 (−4.02, 2.75)	1.717	0.712	
Male <75 (1,510)	1	1.02 (−0.99, 3.03)	1.023	0.320	0.394
	≥2	−4.25 (−6.84, −1.65)	1.322	0.001	
Female <75 (1,836)	1	−0.21 (−2.10, 1.68)	0.963	0.828	0.420
	≥2	0.15 (−1.57, 1.87)	0.874	0.863	

**Figure 2:**
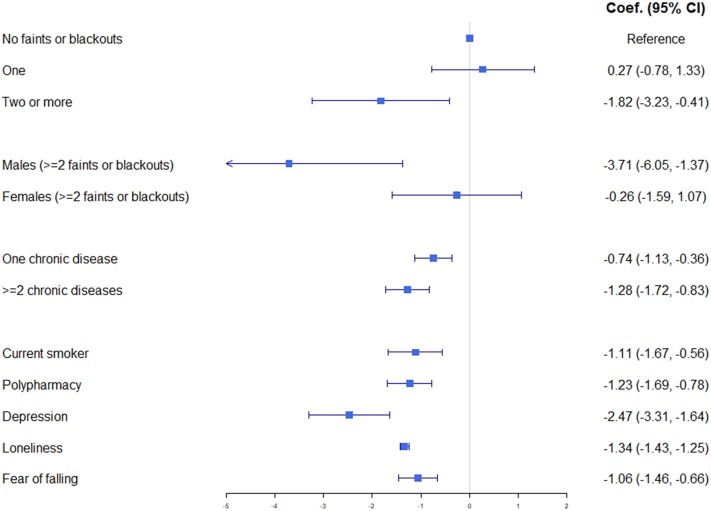
Graphical summary of weighted linear regression Model 3R with coefficient and 95% confidence intervals including those for selected significant covariates for comparison.

These fully adjusted models showed that ≥2 syncopal episodes in the past year were associated with a significantly lower QoL, but when stratified by sex this significance was only seen in males (β = −3.71, 95% CI: −6.05 to −1.37, *p* = 0.002). One recent syncope did not have a significant effect on QoL overall or for either sex when examined individually. Figure 2 shows that two or more syncopal episodes in the past year, in a male population, was observed to have a similar impact on QoL as a current or recent depressive episode and more so than having a single chronic disease from a list including conditions such as chronic lung disease, arthritis, osteoporosis, cancer, Parkinson's disease, liver disease, kidney disease, or any type of incontinence. This relationship would appear to be stronger in younger males.

## Discussion

This study sought to investigate the relationship between syncope and QoL in a large, nationally representative, community-dwelling older population. We observed that syncope was found to have an adverse significant effect on QoL on those who have had a self-reported episode of faint, blackout, or unexplained fall, which is consistent with what has been observed previously in those presenting for assessment at a specialist syncope referral center (13, 14, 16).

We found that a history of a syncopal event, rather than a recent syncopal event, had no significant association with QoL which would indicate that there is not necessarily a prolonged relationship between syncope and QoL. It has been shown previously that QoL improved significantly at 1-year follow up, compared with presentation for syncope (34) but less so for those with recurrent episodes. These results combined suggest it is the frequency of syncopal episodes and how recent they are that affects QoL.

As to why a distant history of a syncopal event, rather than a recent syncopal event, had no significant association with QoL may depend on the type of syncope experienced at different stages of life. Classical VVS, one of the most common subtypes of syncope, usually starts at a young age (35). It is benign and with education about the condition, the precipitating factors and recognition of the prodromal symptoms, the frequency often decreases with age or stops altogether. It would be reasonable that VVS accounted for a significant proportion of syncopal events for the more than 1-in-5 who reported having had a syncopal event in this study. It would also be reasonable that a benign event potentially decades previously would have minimal impact on current QoL.

With regard to whether there were any differences in the relationship observed between syncope and QoL depending on age and sex, a history of a recent syncopal event was found to have an association with QoL for men only. In relation to whether the number of recent syncopal events correlated with QoL, it was found that, while an isolated episode has no effect, ≥2 in the past year has a marked impact on QoL, the degree of which would compare with a current or recent depressive episode.

With regard to the possible mediating effect of FoF on the relationship between syncope and QoL, for those with a recent syncopal event, FoF was observed to have more than twice the effect, in terms of proportion of reduction in the coefficient, for men in the <75 years bracket compared with those ≥75, suggesting it has a higher relative importance with regards QoL for the younger male group. The significance recent syncope had on QoL for women aged ≥75 was lost when FoF was added to Model 3.

Questions raised by our results include why does syncope impact QoL of men more so than women and why does FoF impact younger men more so than old?

Why syncope impacts the QoL of men more than women may be due to the subtype of syncope experienced differing between sexes. For instance, it has been shown (36) that the incidence of cardiac causes of syncope are nearly twice that among men than women (13.2 vs. 6.7%) and that the risk of recurrence was especially high among those with cardiac causes (multivariable-adjusted hazard ratio 30.0; 95% CI: 14.9–60.3) suggesting that, while the prevalence of syncope is higher in women, recurrent syncope is more prevalent in men. Male sex has been shown previously to be a risk factor for recurrent syncope (multivariate hazard ratio 1.18; 95% CI: 1.12–1.24) (37). For those <75, of which 45.1% were male, we observed males accounted for 53.1% of those with ≥2 syncopal episodes in the past year.

Another route in which men may be affected more than women could be the impact syncope may have on social participation which has been shown to play a vital role in healthy aging (38). Strict regulations relating to recent syncope may have direct effects on social participation via restrictions on driving and use of heavy machinery that an episode may have—this may be directly, through its impact on general independence and social participation, or indirectly through subsequent inability to work due to restrictions for those who need to drive as part of their employment. Gaggioli et al. stated that “restrictive measures regarding the resumption of work are often inappropriate given the rarity of recurrences” (39) and while it has been shown that 52% of those presenting to Emergency Departments with syncope are of working age (18–65) only 60% of those employed at the time of syncope returned to their previous job after discharge, with the risk of syncope recurrence highest within 6 months at 9.2% overall (40). Unemployment or working in the home significantly reduced CASP-19 (28).

Driving, rather than being a passenger, is associated with better social participation and well-being whereas relying on lifts was found to be associated with poorer psychosocial well-being (41). The male group were observed to be more likely to be employed (42% for males vs. 33% for females) and there are some professions dominated by one sex or other, such as farming, where farm owners/managers were >88% male in wave 3, while conversely, >99% of those who reported their current employment situation as “looking after home/family” were female. There are other professions such as taxi/bus driving and in the shipping/freight industry which would have a stereotypically male dominated workforce, particularly among older generations, where restrictions on one's license to drive or operate heavy machinery would reduce their ability to work resulting in economic loss, all of which would have a negative effect on QoL. Previous observations in TILDA have shown that income is positively associated with QoL in older age with a mean CASP-12 score of 29.6 for individuals in the highest quintile of household income compared with 26.2 for those in the lowest (42).

With regard to FoF affecting younger men's QoL more so than older men, it has been shown that mental health was a bigger determinant of QoL for a younger age group than older (albeit 50–64 rather than 50–74) with anxiety having a more negative impact than depression when fully adjusted (28). The anxiety around FoF may overlap with poorer mental health as a result of recent syncope potentially unaccounted for with the depression variable in our models. The fact that a large proportion of syncopal episodes never have a confirmed cause may heighten any anxiety and FoF given the level of uncertainty that may bring. As discussed previously, in the age category ≥50, younger men are more likely to have cardiac causes of syncope, more life-threatening causes of syncope and more likely recurrent syncope. Syncope caused by arrhythmias often has little or no prodrome, preventing warning of an event and is more likely to be injurious. All of this may heighten anxiety and FoF when compared with older men or women of any age who have syncope and this may lead to apprehension about returning to certain forms of employment and/or driving, potentially even when unjustified.

Sub-group analyses would suggest that recurrent syncope has an effect on men's QoL, not seen for women, similar to what was observed for recent syncope, with younger men being affected more than older. However, the numbers included in these analyses are small and while they should be interpreted with caution, they merit further consideration and future investigation. Possible reasons for these results may again lie in the subtype of syncope experienced being different between sexes and at different ages and the impact this has on employment status/social participation. Cardiac causes of syncope have been shown to be more prevalent in a more middle-aged pre-retirement group (mean age 56 years) than older, with 10% felt to have ventricular dysrhythmias as the cause of syncope (2). This compared with no syncopal episode at all being attributed to ventricular dysrhythmia in an older group (mean age 87) which would suggest selective survival of individuals without life-threatening dysrhythmias (7). Participants in the Framingham Heart Study (36) with cardiac syncope had lower survival (~55% 5-year survival compared with approximately 85% 5-year survival for the group containing vasovagal, orthostatic, medication-induced and other infrequent causes of syncope) which was consistent with another study that suggested cardiac syncope is associated with increased risk of premature death and cardiovascular events (43). If those with a cardiac cause of syncope are more likely to be younger men, less likely to survive to an older age, and more likely to have recurrent episodes while alive, it could be deduced that their QoL would be worse than older men who are less likely to have cardiac causes of syncope and recurrent syncope.

In conclusion, syncope is a common condition among older adults that has a deleterious effect on QoL, with ≥2 recent syncopal episodes having a particularly adverse impact. FoF is a potential pathway which may both explain some of this association and allow therapeutic interventions by health practitioners. The overall well-being of the general population, rather than just their health, is becoming more of a focus for policy-makers (44). Optimizing health outcomes with primary and secondary prevention/intervention should continue to be first line for healthcare professionals but overall care strategies should also encompass policies that facilitate and sustain social participation of older people in the community. With that in mind, given the results of our investigations, it could be suggested to increase or ear-mark resources to allow for expedition of investigations of syncope where appropriate, such as external loop recorders and echocardiograms for those in employment with the aim of minimizing potential delays before diagnoses and treatment thus reducing the length of driving restrictions and economic losses. Provision of group physical therapy, like cardiac rehabilitation post myocardial infarction, or other similar interventions such as psychological counseling, where appropriate, with the aim of reducing FoF and its impact on QoL may also be advisable.

## Limitations

While TILDA is a longitudinal study the data used here was cross-sectional, so causality cannot be inferred.

The self-report nature of most measures may have introduced a degree of error or bias. While self-reported doctor diagnoses have been shown to be reliable (27) this may not apply to participants' reporting of self-made “diagnoses” relating to falls, blackouts or faints when having not attended a doctor.

Missing information was a limitation − 4,946 participants completed the CASP-12, 76.6% of the original sample size of 6,454, with fewer again (4,843) being included for Models 2 and 3 once those missing data for the remaining covariates of interest were excluded.

While anyone reporting an unexplained fall, blackout, or faint was defined as having had syncope, its subtype is unknown (vasovagal, cardiogenic, pseudo-syncope etc.) and the outcome may be different for different classes of syncope. It has been shown that those with an underlying neurological or psychogenic cause of syncope have a poorer QoL (34) and it is possible that participants with a pre-existing poor QoL somatise with consequential psychogenic syncope.

The CAPI had no question relating to history of unexplained falls beyond the last year or since last interview. Therefore, while those reporting recent syncope were classified as anyone with a positive answer to recent unexplained fall, blackout or faint, those reporting previous syncope were classified as those with a positive answer to history of blackout or faint. They are therefore not directly comparable.

Similarly, while the CAPI quantified the burden of recent syncopal events by asking about the number of recent faints or blackouts, the burden of unexplained falls was not quantified individually, instead being quantified within the number of falls as a whole. The recurrent syncope predictor variable in our models includes only those reporting a blackout or faint in the past year and not those with unexplained falls, so this would need to be considered when interpreting results and again it is not directly comparable with our recent syncope analyses.

As mentioned in the discussion, while it was observed that the burden of recent faints or blackouts had a significant relationship with QoL with a marked decrease in QoL for men having 2 or more events in the past year, it should be noted that the absolute numbers in the different categories are low (when stratified by sex and age having excluded those with unexplained falls and participants missing data on all covariates of interest). A total of 44 participants reported ≥2 syncopal episodes in the past year, 21 male (17 <75), 23 female (15 <75), therefore particularly the analyses for those ≥75 should be interpreted with caution.

The mean age of this group at baseline was 64.8 which is approaching the age when prevalence of syncope begins to rise sharply peaking around 70 years of age (45). Therefore, it would be valuable to follow up this cohort of participants in further waves and evaluate their QoL as syncope becomes more prevalent.

## Data Availability Statement

TILDA datasets are in a publicly accessible repository: The datasets analyzed for this study can be found at http://www.ucd.ie/issda/.

## Ethics Statement

Ethical approval was obtained from the Faculty of Health Science Research Ethics Committee at Trinity College Dublin. Informed written consent was obtained from all participants. The patients/participants provided their written informed consent to participate in this study.

## Author Contributions

RK is the Principal Investigator and project coordinator of the TILDA study. KM, MW, and RK designed the study and analyzed the data. KM wrote the initial draft of the manuscript. MW, RR, and RK critically revised the manuscript. All authors had responsibility for accuracy of the final content and read and approved the final manuscript.

### Conflict of Interest

The authors declare that the research was conducted in the absence of any commercial or financial relationships that could be construed as a potential conflict of interest.

## Non-standard abbreviations

CAPI: Computer Assisted Personal Interview
CASP: Control, Autonomy, Self-realization and Pleasure
CIDI: Composite International Diagnostic Interview
FoF: Fear of falling
QoL: Quality of life
SCQ: Self-Completion Questionnaire
TILDA: The Irish Longitudinal Study of Aging
UCLA: University of California, Los Angeles
VVS: Vasovagal syncope.
